# Electron Paramagnetic Resonance Gives Evidence for the Presence of Type 1 Gonadotropin-Releasing Hormone Receptor (GnRH-R) in Subdomains of Lipid Rafts

**DOI:** 10.3390/molecules26040973

**Published:** 2021-02-12

**Authors:** Tilen Koklič, Alenka Hrovat, Ramon Guixà-González, Ismael Rodríguez-Espigares, Damaris Navio, Robert Frangež, Matjaž Uršič, Valentina Kubale, Ana Plemenitaš, Jana Selent, Marjeta Šentjurc, Milka Vrecl

**Affiliations:** 1 Laboratory of Biophysics, Department of Condensed Matter Physics, Jožef Stefan Institute, 1000 Ljubljana, Slovenia; tilen.koklic@ijs.si (T.K.); marjeta.sentjurc@ijs.si (M.Š.); 2 Veterinary Faculty, Institute of Preclinical Sciences, University of Ljubljana, Gerbičeva 60, 1000 Ljubljana, Slovenia; alenka.hrovat@scarsdalevets.com (A.H.); robert.frangez@vf.uni-lj.si (R.F.); matjaz.ursic@vf.uni-lj.si (M.U.); valentina.kubaledvojmoc@vf.uni-lj.si (V.K.); 3 Research Programme on Biomedical Informatics (GRIB), Department of Experimental and Health Sciences, Hospital del Mar Medical Research Institute (IMIM), Pompeu Fabra University (UPF), 08003 Barcelona, Spain; ramon.guixa@psi.ch (R.G.-G.); ismael.rodriguez@upf.edu (I.R.-E.); damarisnavio@gmail.com (D.N.); jana.selent@upf.edu (J.S.); 4 Laboratory of Biomolecular Research, Paul Scherrer Institute (PSI), 5232 Villigen, Switzerland; 5 Condensed Matter Theory Group, PSI, 5232 Villigen, Switzerland; 6 Faculty of Medicine, Institute of Biochemistry and Molecular Genetics, University of Ljubljana, 1000 Ljubljana, Slovenia; ana.plemenitas@mf.uni-lj.si

**Keywords:** 7TM receptors, type 1 gonadotropin-releasing hormone receptor, plasma membrane, electron paramagnetic resonance (EPR), lipid rafts

## Abstract

This study investigated the effect of type 1 gonadotropin releasing hormone receptor (GnRH-R) localization within lipid rafts on the properties of plasma membrane (PM) nanodomain structure. Confocal microscopy revealed colocalization of PM-localized GnRH-R with GM_1_-enriched raft-like PM subdomains. Electron paramagnetic resonance spectroscopy (EPR) of a membrane-partitioned spin probe was then used to study PM fluidity of immortalized pituitary gonadotrope cell line αT3-1 and HEK-293 cells stably expressing GnRH-R and compared it with their corresponding controls (αT4 and HEK-293 cells). Computer-assisted interpretation of EPR spectra revealed three modes of spin probe movement reflecting the properties of three types of PM nanodomains. Domains with an intermediate order parameter (domain 2) were the most affected by the presence of the GnRH-Rs, which increased PM ordering (order parameter (S)) and rotational mobility of PM lipids (decreased rotational correlation time (τc)). Depletion of cholesterol by methyl-β-cyclodextrin (methyl-β-CD) inhibited agonist-induced GnRH-R internalization and intracellular Ca^2+^ activity and resulted in an overall reduction in PM order; an observation further supported by molecular dynamics (MD) simulations of model membrane systems. This study provides evidence that GnRH-R PM localization may be related to a subdomain of lipid rafts that has lower PM ordering, suggesting lateral heterogeneity within lipid raft domains.

## 1. Introduction

The discovery of lipid rafts and other domains of the plasma membrane (PM) has led to a re-evaluation of the classical Singer-Nicolson fluidic mosaic model, which described cell membranes as a more or less homogeneous phospholipid bilayer [1]. Currently, there is a growing consensus that the potential for lateral segregation of PM is based on the preferential association among sphingolipids, sterols, and specific proteins, which underlies the raft concept of PM subcompartmentalization (reviewed in [2]). Lipid rafts (also called lipid nanodomains) in the PM outer leaflet have been defined as small (10–200 nm), heterogeneous, and highly dynamic domains enriched with glycosphingolipids and cholesterol that compartmentalize cellular processes [3,4]. The tight packing of the saturated acyl chains of sphingolipids and the presence of cholesterol appear to result in a domain that is thicker, more ordered, and less fluid than the surrounding phosphatidylcholine-enriched PM environment. Factors that determine the partitioning/targeting of proteins in lipid rafts include various types of lipidation (e.g., myristoylation and palmitoylation), glycophosphatidylinositol (GPI) linkage, and covalent binding of cholesterol (reviewed in [5,6]). In addition, protein interactions with scaffolding raft-resident proteins—e.g., caveolin—transmembrane domain (TM) length, oligomerization, and preferential interaction of protein segments at the membrane interface with certain lipid components could also be responsible for lipid raft association (reviewed in [5,7,8,9]).

Many membrane receptors, including seven-transmembrane receptors (7TMRs), also known as G-protein coupled receptors (GPCRs), are not uniformly distributed in the PM but can either be constitutively localized in lipid rafts or have the ability to move in and out of these nanodomains [10,11]. Several 7TMRs have been experimentally shown to be palmitoylated at conserved carboxyl-terminal cysteine residues (reviewed in [12]), which may control the distribution/targeting of proteins in lipid rafts. Additional factors governing 7TMR targeting to lipids rafts could include the presence of specific cholesterol binding sites on the 7TMRs (reviewed in [13]), or specific lipid species [14]. A computational study also suggested the existence of interdependence between the lateral domain structure of the PM and the 7TMR dimerization state [15].

The mammalian type I gonadotropin-releasing hormone receptor (GnRH-R) is structurally unique among 7TMRs, including other GnRH-Rs expressed in non-mammalian and some primate species, in that its cytoplasmic carboxyl-terminal tail is only one to two amino acids long and therefore lacks putative palmitoylation sites [16]. Nevertheless, this receptor localizes constitutively and almost exclusively to low-density detergent-resistant membrane fractions representing lipid rafts in (i) immortalized pituitary gonadotrope-derived αT3-1 and LβT-2 cells [16,17,18,19], (ii) whole mouse pituitary [18], (iii) cultured bovine gonadotrophs [20], and (iv) heterologous Chinese hamster ovary (CHO) cells [18]. Its PM localization is also independent of hormone treatment [18]. Fusion of the palmitoylated carboxyl-terminal tail of the luteinizing hormone receptor (LH receptor), which is normally excluded from lipid rafts, to GnRH-R alters its PM localization [21], whereas fusion of the palmitoylated carboxyl-terminal tail of non-mammalian GnRH-Rs preserves its lipid raft localization [22]. It can therefore be assumed that the constitutive localization in lipid rafts is not due to the absence of a carboxyl-terminal tail, but rather is a conserved feature of non-tailed and tailed GnRH-Rs. The localization of GnRH-R within lipid rafts was also functionally supported by (i) cross-talk between the GnRH-R and the glucocorticoid receptor in LβT-2 mouse pituitary cell line that involves co-localization with lipid rafts [19] and (ii) cholesterol depletion, which prevents GnRH-R-mediated activation of the extracellular signal-regulated kinase (ERK) pathway [18,23] and induces gonadotropin release in ovine pituitary and LβT-2 cells [24].

Considering the abovementioned evidence for localization of GnRH-R within lipid rafts, we hypothesized that its compartmentalization influences PM nanodomain properties. Two pharmacologically well-characterized continuous cell model systems were used to test this assumption: gonadotrope-derived αT3-1 cells expressing endogenous mouse GnRH-R [25], and heterologous HEK-293 cell lines stably expressing either WT or HA-tagged rat GnRH-R [26], both of which express GnRH-R at levels lower than or equivalent to primary gonadotropes (reviewed in [27]). Electron paramagnetic resonance spectroscopy (EPR) has been used to study the lateral structure of PM nanodomains. EPR, in combination with computer simulation of the spectra and the GHOST condensation procedure, is very informative in distinguishing different PM nanodomains in living cells and provides insight into the physical properties of PM, such as fluidity and domain structure [28]. The spin probe MeFASL(10,3) used has the nitroxide group positioned on the 5th carbon atom of the fatty acid alkyl chains [29] and thus reports the membrane domains and their fluidity properties in the upper part of the membrane layers (close to the surface of the lipid bilayer). Moreover, oxy-redox systems in cell organelles and cytoplasm reduce spin probes to hydroxylamines, which are undetectable by EPR, so that only spin probes in the PM contribute to the EPR signal [30,31], allowing us to detect changes in the PM lateral domain properties due to the specific localization of GnRH-R.

## 2. Results

### 2.1. PM Localization of GnRH-R in HEK-293 Cells

**Figure 1:**
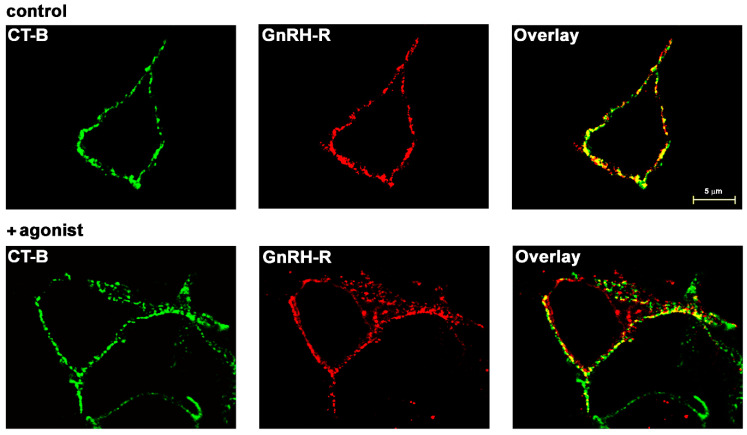
Colocalization of GnRH-R with glycosphingolipid GM_1_-enriched raft-like domain regions of the PM in HEK-293 cells. Live HEK-HAGnRH-R cells were first labeled with the green-fluorescent Alexa Fluor 488 conjugate of CT-B. Cells were then fixed and immunostained for HA-tagged GnRH-R using primary anti-HA monoclonal antibody and then a TRITC-conjugated secondary antibody. Sequentially acquired confocal images show GM_1_-enriched raft-like PM subdomains and a GnRH-R distribution pattern and colocalization in control (upper panels) and agonist treated (D-Trp^6^-GnRH; 1 μM for 1 h at 37 °C) cells (lower panels). Green color indicates lipid rafts; red is HA-tagged GnRH-R. Merged images show the overlay of two channels; yellow/orange is the overlapping region indicating colocalization of GnRH-R and GM_1_-enriched raft-like domains. Note that PM-localized GnRH-R colocalizes with GM_1_-enriched raft-like PM subdomains in control (unstimulated) and agonist-treated cells.

Confocal microscopy showed predominant PM localization of HAGnRH-R and its high colocalization (Pearson’s correlation coefficient 0.70) with fluorescent cholera toxin subunit B (CT-B) conjugate, a marker for ganglioside GM_1_-enriched raft-like PM subdomains, in control (unstimulated) cells (Figure 1; upper panels). Receptor stimulation with the agonist D-Trp6-GnRH (1 μM; 1 h at 37 °C) resulted in partial redistribution of cellular immunostaining, indicating internalization of GnRH-R and also a lower degree of colocalization (Pearson’s correlation coefficient 0.44). However, most of the PM-localized receptor was still colocalized with the CT-B conjugate (Figure 1; lower panels). CT-B conjugate partitioning in lipid rafts was also shown by sucrose gradient (5%/35%/85%) centrifugation (Supplementary Figure S1). The predominant localization of GnRH-R in the glycosphingolipid GM_1_-enriched raft-like domain regions of the PM in αT3-1 cells has been reported previously [17,18].

### 2.2. Functional Characterization of GnRH-R in HEK-293 Cells—Effect of Cholesterol Depletion

**Figure 2:**
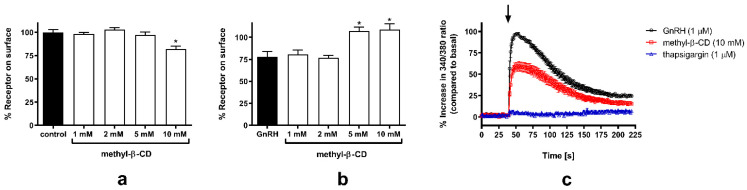
Effect of methyl-β-CD treatment on GnRH-R surface expression, agonist-induced internalization, and [Ca^2+^]_i_ in HEK-HAGnRH-R cell line. (**a**) ELISA was performed to determine the effect of increasing concentrations of methyl-β-CD treatment (from 1 to 10 mM; 1 h at 37 °C) on the level of surface-expressed HAGnRH-R. Measurements were performed as described in Section 4, Material and Methods. Data (mean ± S.E.) are expressed as a percentage of the value obtained in untreated (control) cells from a single experiment performed in triplicate and are representative of a total of three independent experiments. * *p* < 0.05 versus control. (**b**) Effect of methyl-β-CD treatment on the agonist-induced HAGnRH-R internalization. HEK-HAGnRH-R cells were pretreated with assay medium containing increasing concentrations of methyl-β-CD (from 1 to 10 mM; 1 h at 37 °C), and the methyl-β-CD concentration was maintained during ligand treatment (1 μM D-Trp^6^-GnRH; 30 min at 37 °C). The levels of surface-expressed receptors after agonist (GnRH) treatment in the absence or presence of methyl-β-CD. Data represent the mean ± S.E. of three independent experiments performed in triplicate. * *p* < 0.05 versus GnRH-treated cells. (**c**) Time-dependent changes in F_340_/F_380_ ratio in HEK-HAGnRH-R cells upon exposure to 1 μM D-Trp^6^-GnRH alone or 1 μM D-Trp^6^-GnRH after pretreatment with 10 mM methyl-β-CD (methyl-β-CD) or 1 µM thapsigargin in DPBS. Representative time-course data from one of at least three experiments performed in triplicate are shown. The time point of GnRH injection is marked by an arrow. Note the decreased response after pretreatment with methyl-β-CD, whereas thapsigargin almost completely prevents the increase in [Ca^2+^]_i_.

To provide supporting functional evidence for the localization of GnRH-R in raft-like PM subdomains, we next tested the effect of cholesterol depletion on GnRH-R surface expression, agonist-induced internalization, and intracellular Ca^2+^ activity [Ca^2+^]_i_. GnRH-R is preferentially coupled to phosphoinositidase C via the Gα_q_/G_11_ family of G-proteins and its stimulation by agonist induces the production of inositol phosphates (IPs), which results in the elevation of [Ca^2+^]_i_ (reviewed in [32]) and induces very slow time-dependent loss of the cell surface receptors [33,34]. First, we tested the effect of increasing concentrations of methyl-β-cyclodextrin (methyl-β-CD; 1–10 mM), leading to a progressive reduction in cholesterol content, on HAGnRH-R surface expression. Concentrations up to 5 mM had no detectable effect on receptor surface expression, whereas in the presence of 10 mM methyl-β-CD an approximately 18% decrease in GnRH-R surface expression was observed by ELISA (Figure 2a). The GnRH-R internalization process was completely suppressed in the presence of 5 or 10 mM methyl-β-CD, while lower concentrations of methyl-β-CD had no obvious effect (Figure 2b). The GnRH-induced increase in [Ca^2+^]_i_ was about 40% lower in the presence of 10 mM methyl-β-CD. The agonist-induced increase in [Ca^2+^]_i,_ originated from internal stores, as shown by the thapsigargin effect (Figure 2c). For further experiments, 10 mM methyl-β-CD was chosen; it was previously shown that at this concentration, cholesterol content was reduced by about 30% [35], while the decrease in surface GnRH-R expression was less than 20%.

### 2.3. PM Properties of GnRH-R Expressing Cells

**Figure 3:**
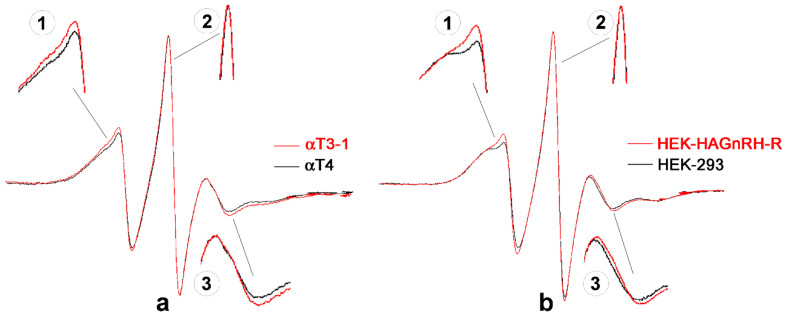
EPR spectra of the lipophilic spin probe MeFASL (10,3) in the PM of immortalized pituitary gonadotrope and heterologous cell lines. (**a**) EPR spectra of immortalized pituitary gonadotrope cell line (αT3-1 cells; red line) and control gonadotrope progenitor cells lacking GnRH-R (αT4 cells, black line). (**b**) EPR spectra of HEK-HAGnRH-R cell line stably expressing HA-tagged GnRH-R (red line) and of control, untransfected HEK-293 cells (black line). Insets 1, 2, 3, magnified portions of spectra showing the difference in line shape between cell lines lacking receptors and GnRH-R expressing cell lines.

Since the localization of GnRH-R in the PM raft-like domains was also demonstrated in HEK-293 cells and supported by the observed effect of cholesterol depletion on GnRH-R internalization and [Ca^2+^]_i_, we next investigated the effect of receptor-lipid raft association on PM properties by EPR spectroscopy using MeFASL(10,3) as a spin probe. The EPR spectra for PM of GnRH-R expressing cell lines (red lines), i.e., αT3-1 and HEK-HAGnRH-R, and for their corresponding controls (black lines; αT4 and HEK-293) are shown in Figure 3a,b, respectively. Portions of the spectra showing a difference in line shape between cell lines that lack receptors and GnRH-R expressing cell lines are shown magnified (Figure 3; insets 1, 2, 3). From inserts 1 and 3 it can be seen that the spin probe moves more unevenly in the PM of GnRH-R expressing cell lines (the spin probe is less restricted in its movement) because the peaks of the EPR spectra are narrower and therefore higher. For a more detailed interpretation of EPR results, we used a computer simulation of the EPR spectra in combination with GHOST condensation procedure.

### 2.4. Computer Simulation of EPR Spectra

**Figure 4:**
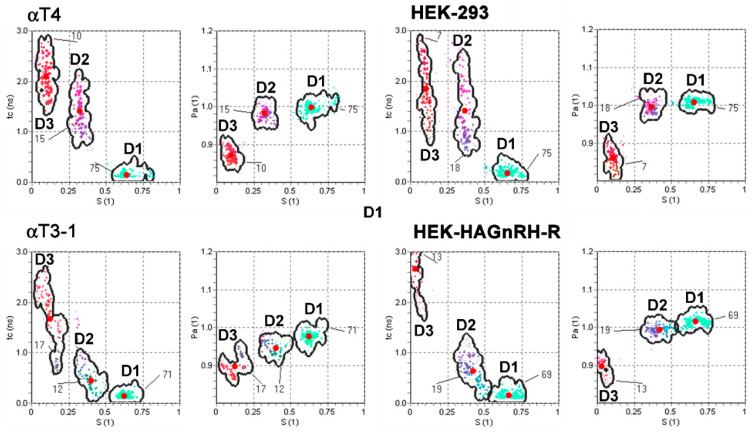
Effect of GnRH-R expression on the PM domain structure of pituitary gonadotrope and heterologous cell line. S-τc (order parameter vs. rotational correlation time) and S-p_A_ (order parameter vs. polarity correction factor) GHOST diagrams of EPR spectral parameters of motional modes of the spin probe in the PM of gonadotrope progenitors lacking endogenous GnRH-R (αT4 cells), immortalized pituitary gonadotrope cell line (αT3-1 cells), untransfected HEK-293 cells and HEK-293 cells stably expressing HA-tagged GnRH-R (HEK-HAGnRH-R). The red dots are the average values of the spectral parameters obtained from multiple runs of the HEO-optimization procedure, yielding the best fits to the experimental spectra. The boundaries of each group determine the interval in which the solutions can be obtained, and the numbers correspond to the proportion of each domain type (%). GHOST diagrams are representative of at least four independent experiments. Domain 1 (D1), domain 2 (D2), domain 3 (D3).

**Figure 5:**
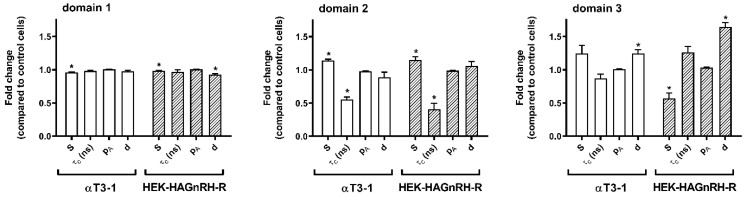
Order parameters (S), rotational correlation times (τc), polarity correction factor (pA), and the proportions (d) of the different domain types in the pituitary gonadotrope and heterologous GnRH-R expressing cell line. The parameters of the best fit of the calculated to the experimental spectra were determined from the GHOST diagrams as a value at which the density of the solutions is maximal (red dots in the GHOST diagram—Figure 4). Order parameters (S), rotational correlation times (τc), polarity correction factor (pA), and the proportions (d) of the different domain types. The data (mean ± S.E.) from at least four independent computer simulations are expressed as the relative change of each parameter obtained in the GnRH-R expressing cell lines (αT3-1 and HEK-HAGnRH-R) compared to the corresponding control receptor non-expressing cell lines (αT4 and HEK-293); * *p* < 0.05.

To gain better insight into the changes in PM domain properties, a computer simulation of EPR spectra was performed, taking into account that the PM is heterogeneous and consists of regions with different fluidity properties [36,37]. Good agreements were obtained with the experimental spectra considering that the spectra are composed of three spectral components. This indicates that the PM of studied cell lines is heterogeneous and consists of several regions with different modes of spin-probe motions. It should be stressed that the lateral motion of the spin probe within the membrane is slow on the time scale of the EPR spectra [37,38]. Each spin probe molecule, therefore, reflects the motional properties of its nearest surrounding on the nanometer scale. EPR spectral contributions of all spin probe molecules located in membrane regions with the same properties give one spectral component. These membrane regions are referred to as a certain type of membrane domain, with dimensions of the order of magnitude of several nanometers. Several small regions with the same physical properties cannot be distinguished from a few large regions and determine the spin probe motion pattern, regardless of their location in the membrane. This also means that the EPR does not necessarily directly reflect the macroscopic properties of the membrane or large membrane domains, but rather the membrane superstructure on the nanometer scale (membrane nanodomains). Representative GHOST diagrams for the EPR spectra measured at 20 °C are presented in Figure 4. The parameters (order parameter (S), spin probe rotational correlation time (τc), polarity correction factor (pA) and the portion of different domain types (d)) were obtained from the groups in the GHOST diagrams where the density of the solutions is maximal (red dots in the GHOST diagram in Figure 4) and are summarized in Figure 5. In the GHOST diagrams, S vs. τc and S vs. pA are plotted for one representative experiment (the results for the other three experiments are comparable). As shown in Figure 4, three regions with different modes of spin probe motion were detected in the PM of both control (αT4 and HEK-293) and GnRH-R expressing cell lines (αT3-1 and HEK-HAGnRH-R), which corresponded to three types of membrane nanodomains (domain 1 with the highest order parameter; domain 2 with intermediate order parameter; domain 3 with the lowest order parameter). However, in GHOST diagrams of GnRH-R expressing cell lines, i.e., αT3-1 and HEK-HAGnRH-R, the domain type with an intermediate order parameter (S = 0.37 and 0.41 for αT3-1 and HEK-HAGnRH-R cells, respectively) changed the most and approached the group representing the most highly ordered domain 1 (compare Figure 4 lower and upper panels). Compared to the corresponding controls, the calculated parameters showed the following similarities in the PM properties of both GnRH-R expressing systems: (i) decrease in the order parameters (S) of domains 1; (ii) increase in the order parameters (S) and decrease in the rotational correlation time (τc) of domain 2; and (iii) an increase in the membrane area (d) of domain 3 (Figure 5, where the relative changes in the mean EPR parameters with respect to the control are shown together with the mean errors of the fits). A decrease in membrane area (d) of domain 1 and order parameters (S) of domain 3 were also observed in HEK-HAGnRH-R cells compared to HEK-293 cells (Figure 5).

Since previous studies have suggested that epitopes and chimeric tags may affect the localization of GnRH-R [39], the PM lateral domain structure of the HEK-293 stable cell line expressing WT rat GnRH-R (HEK-GnRH-R) was also examined. The EPR spectra and GHOST diagrams of HEK-GnRH-R and HEK-HAGnRH-R cells were comparable, indicating that the N-terminally positioned HA epitope tag had no detectable effect on GnRH-R PM-localization (Supplementary Figure S2). There was also no detectable effect of agonist treatment on the lateral domain structure of GnRH-R expressing cell lines, as the EPR spectra for the PM of untreated (control) and agonist-treated HEK-HAGnRH-R cells showed no obvious differences (Supplementary Figure S3).

### 2.5. Properties of the PM—Effect of Cholesterol Depletion

**Figure 6:**
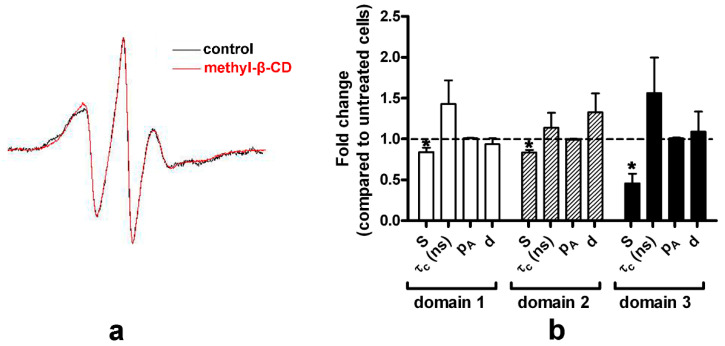
Effect of methyl-β-CD treatment on PM properties of the HEK-HAGnRH-R cell line. (**a**) Comparison of EPR spectra of the lipophilic spin probe MeFASL (10,3) in the PM of untreated (control; black line) and methyl-β-CD (10 mM, 60 min at 37 °C; red line) HEK-HAGnRH-R cells. The spectra shown are the sum of spectra from at least four independent experiments. (**b**) Order parameters (S), rotational correlation times (τc), polarity correction factor (pA), and the proportions (d) of the different domain types in HEK-HAGnRH-R cells. The parameters of the best fit of the calculated to the experimental spectra were obtained from GHOST diagrams. Results (mean ± S.E.) of four independent computer simulations are expressed as the relative change of each parameter obtained in cells treated with methyl-β-CD compared to untreated (control) HEK-HAGnRH-R cells * *p* < 0.05.

**Figure 7:**
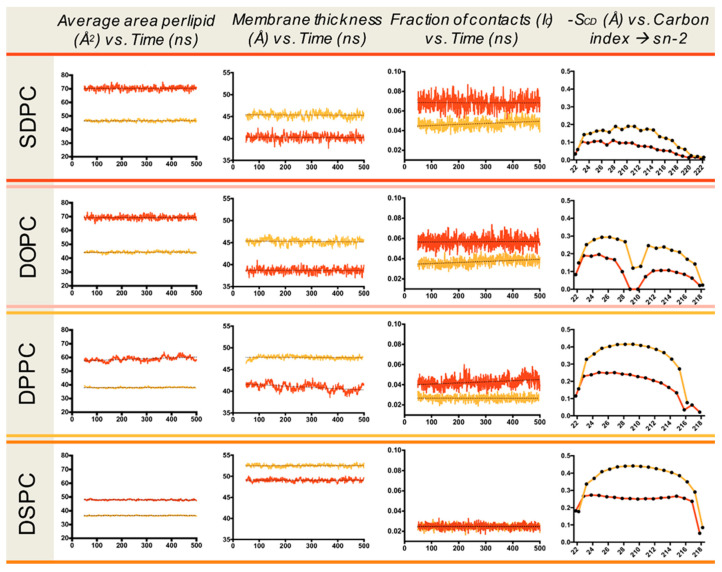
MD simulation of membrane local properties—impact of cholesterol. Averaged properties for each lipid species with (33%; orange line) and without cholesterol (red line). S_CD_ order parameters, membrane thickness, lipid interdigitation, and area per lipid were obtained using MEMBPLUGIN [41].

PM is enriched in raft-forming lipids (cholesterol and sphingolipids) and cholesterol typically presents 30–40 mol% of PM lipids [40]. The EPR spectra for the PM of untreated (control) and methyl-β-CD treated HEK-HAGnRH-R cells are shown in Figure 6a and the derived parameters are summarized in Figure 6b. Compared to the untreated HEK-HAGnRH-R cells, the calculated parameters showed the following effects of methyl-β-CD treatment on PM properties: (i) decrease in the order parameters (S) of all three domain types and (ii) tendency for a variable increase in the rotational correlation time (τc) of domain 1, 2, and 3 (Figure 6b). The influence of cholesterol was further investigated on model membranes by MD simulations. In these simulations, the inclusion of 33% cholesterol in phospholipid bilayers lead to a condensation effect, represented by an increase in membrane thickness and a decrease in average area per lipid (Figure 7). This condensation effect clearly decreases membrane fluidity (i.e., more rigid lipid acyl chains), as shown by the increase in lipid order parameters (S_CD_) (Figure 7). Likewise, more rigid lipid tails can interdigitate less, thus showing a lower fraction of inter-leaflet contacts (Figure 7).

## 3. Discussion

Partitioning/clustering of membrane proteins in specific PM nanodomains can lead to reorganization of nanodomain composition and/or their physical properties. Using confocal microscopy, this study first demonstrated a preferential colocalization of PM-localized GnRH-R with the GM_1_-enriched raft-like PM subdomains in HEK-293 cells, which was independent of agonist stimulation. Previous studies have reported the same localization in different cell model systems and the whole mouse pituitary [16,17,18,19,20]. Because the size of lipid rafts is smaller than the resolution of confocal microscopy (~250 nm lateral resolution limit), interpretation of colocalization data should be done with caution. Supporting functional evidence for the involvement of lipid rafts in GnRH-R regulation was provided by cholesterol depletion with methyl-β-CD. Surface expression of GnRH-R in HEK-293 cells was not largely affected by treatment with methyl-β-CD, consistent with data obtained with cholesterol-depleted αT3-1 cells [18]. In contrast, its agonist-induced internalization and [Ca^2+^]_i_ depended on the integrity of lipid rafts. The effect of cholesterol depletion on Ca^2+^ signaling rather than cell surface density was also reported for another Gα_q/11_-coupled 7TMR, namely the neurokinin-1 receptor (NK1R) [42]. Chicken GnRH-R internalization was also inhibited in both methyl-β-CD and filipin cholesterol-depleted cells [43]. It has also been shown that the residence of GnRH-R in lipid rafts is a prerequisite for its ability to activate the ERK signaling pathway (reviewed in [23]).

Lipid rafts are very difficult to study as the methodology for detection must either be extremely sensitive at nanometer and millisecond scales or differences between probes amplified by cellular machinery [3]. We employed EPR spectroscopy in combination with computer simulation of the spectra to monitor how a cell adapts its lateral domain organization to GnRH-R clustering in lipid rafts at receptor densities close to physiological concentrations of GnRH-R in the anterior pituitary (reviewed in [27]). It should be emphasized that domain types obtained by EPR/GHOST do not necessarily correspond to biochemically characterized lipid rafts. Employing the GHOST condensation routine, we were able to define three types of membrane nanodomains with different ordering and dynamics. The most ordered nanodomains (domain 1) predominated, followed by domain 2 and 3. This is in agreement with recent data suggesting that Lo (ordered membrane) domains may predominate over less ordered Ld (non-raft) domains, although the opposite was originally suggested (reviewed in [9]). Immunocytochemical evidence for GnRH-R lipid raft localization correlated with only a small decrease in the order parameters (S) of domain 1 (the most ordered nanodomains) in both GnRH-R expressing cell lines (Figure 5). Instead, we found pronounced changes for both cell lines in properties of the nanodomains with an intermediate order parameter (domain 2). This was manifested in GHOST diagrams of GnRH-R expressing cell lines (Figure 4) in which domain 2 approaches domain 1 with a characteristic decrease in the rotational correlation time (τc) and an increase in the order parameter (S) in domain 2. The τc of spin label motion is related to the time lapse between subsequent molecular collisions, which affect the rotational moment and should correlate with the free rotational space. This implies that a spin label in a more confined environment can maintain its rotational moment for a shorter time, i.e., it has a shorter correlation time, and vice versa: a spin label that is spatially less restricted remembers its motional momentum for a longer time, i.e., it has a longer τc [44]. A decrease in the τc in domain 2, therefore, suggests that the spin probe is trapped between some rigid structures such as cholesterol and receptors. Membrane protein clustering in the lipid membrane can manifest either by the disappearance of membrane domains or by the emergence of new domains with intermediate dynamic properties [45] so that domains can operate as protein concentrators or isolators. The inclusion of 7TMRs in the biochemically characterized lipid rafts seems to coincide with an alteration in domain 2. Interestingly, this alteration of domain 2 results in its interconnection with domain 1 according to the GHOST diagrams (Figure 4). Based on previous reports [6,46], domain 2, which is favored by lipid raft associated 7TMRs, could be either regarded as areas of lipid rafts with a lower order parameter or distinctive regions at the raft/membrane interface in which a subset of proteins becomes locally enriched. This corroborates with previous reports showing that (i) raft-associated TM proteins are excluded from the ordered (Lo) phase in a model membrane system [47] and (ii) the GM_1_ phase in PM spheres is distinct from the Lo phase; it displays a considerably lower order and supports the inclusion of raft TM proteins [48]. The motional pattern of MeFASL(10,3) reflects the characteristics of lateral domain types on the nanometer scale close to the PM surface and provides information about an area that could be more than 10 times smaller than the estimated diameter of lipid rafts, bearing in mind the convergence of estimates for the diameter of lipid rafts being 10 to 200 nm [2]. It is therefore very likely that the motional pattern of MeFASL(10,3) not only varies between different domain types but also within a particular biochemically characterized domain. Besides this, atom-scale computer simulations have also proposed nanoscale lateral heterogeneity within raft domains with different chain ordering [46].

Fluorescence photobleaching recovery (FRP) data have previously revealed a fast lateral diffusion (~1.2 × 10^−9^ cm^2^/sec) of the unoccupied murine GnRH-R C-terminally tagged with green fluorescent protein in the PM of αT3-1, CHO and COS-1 cells [49]. The binding of the agonist to GnRH-R slowed down the rate of lateral movement and reduced the fraction of mobile receptors in the PM [49]. Agonist-induced effects observed by FRP, which were attributed to the formation of self-associated receptor complexes [50], could not be detected by EPR. However, this is not always the case, since we were able to detect changes between control and agonist treated HEK-293 cells stably expressing NK1-R [51]. In contrast to the GnRH-R, NK1-R displays extensive and very rapid agonist-induced internalization; approximately 80% of the receptors are endocytosed after 10 min of agonist stimulation [51]. It could therefore be assumed that agonist-induced changes in the GnRH-R surface expression/localization are too small to result in detectable changes in PM domain structure when studied by EPR. Previous reports have also revealed that both GnRH-R and NK1-R concentrate in microdomains representing only a very small proportion of the total surface area of the PM [17,52]. For NK1-R, it was estimated to concentrate in cholesterol-enriched domains with an estimated diameter of ~10 nm, which represents ~1% (0.8%–2.5% depending on the receptor density) of the total surface area of the PM [17,52].

In mammalian cell membranes, cholesterol is regarded as a major modulator of lipid lateral segregation and packing density; however, differences in the structure of the lateral domains of PM leaflets could not be detected by current methods, although they probably exist, considering that only the outer PM leaflet is rich in sphingolipids and cholesterol [3]. We examined the effect of methyl-β-CD treatment (cholesterol depletion) on the lateral domain organization of HEK-HAGnRH-R cells. EPR spectroscopy and the GHOST condensation routine showed that the treatment of cells with 10 mM methyl-β-CD leads to an overall reduction in the PM order, as demonstrated by a decrease in the order parameters (S) in all three domain types. The most prominent decrease in the PM order was observed in domain 3, which already has the lowest order parameter. In support of our EPR/GHOST observations, desorption of cholesterol to β-cyclodextrin showed that cholesterol removal from sphingomyelin monolayers is less efficient than removal from less ordered acyl chain-matched phosphatidylcholine monolayers, due to its higher affinity for sphingomyelin over chain-matched phosphatidylcholine [53]. MD simulations of the model membrane systems also showed changed local biophysical properties of simulated membranes in the presence of cholesterol.

In summary, our results show that the localization of GnRH-R in the GM_1_-enriched raft-like PM subdomains, as determined by immunocytochemical methods, correlates primarily with a change in domain 2 that may be related to the region of lipid rafts that has a lower order, providing evidence for lateral heterogeneity within lipid raft domains in living cells. Interference of lipid raft domains by cholesterol depletion provided additional functional evidence for the importance of these PM domains for the agonist-induced GnRH-R internalization and Ca^2+^ signaling. To provide further conclusive evidence for the existence/heterogeneity of lipid rafts, the use of state-of-the-art techniques is required, i.e., super-resolution fluorescence microscopy in combination with novel fluorescent probes for specific membrane domains and cryo-electron microscopy, which allow visualization of subcellular structures on a nanoscale level in vivo and in situ.

## 4. Materials and Methods

### 4.1. Materials

Human embryonic kidney (HEK-293) cells were obtained from the European Collection of Cell Cultures (Porton Down, Salisbury, UK). Stable cell lines expressing WT and N-terminally hemagglutinin (HA)-tagged rat GnRH-R, hereafter referred to as HEK-GnRH-R and HEK-HAGnRH-R, respectively, were kindly provided by the MRC Human Reproductive Sciences Unit, Edinburgh, UK. Immortalized pituitary gonadotrope cell line (αT3–1 cells) and gonadotrope progenitors lacking endogenous GnRH-R (αT4 cells), established by Windle et al. [54], were kindly provided by Professor P. Mellon (University of California, San Diego, La Jolla, CA, USA). All tissue media and reagents were obtained from either Gibco Invitrogen (Carlsbad, CA, USA) or Sigma Aldrich (St. Louis, MO, USA) unless otherwise stated. In addition, anti-HA high affinity rat monoclonal antibody (clone 3F10) was obtained from Roche Diagnostics GmbH (Mannheim, Germany). Anti-rat horseradish peroxidase (HRP)-conjugated and TRITC-conjugated secondary antibodies were from Sigma Aldrich (St. Louis, MO, USA). Vybrant^®^ Alexa Flour^®^ 488 Lipid Raft Labeling Kit was from Molecular Probes Europe BV (Leiden, The Netherlands). Thapsigargin and Fura-2 AM were from Molecular Probes (Eugene, OR, USA) and sucrose from Merck (Darmstadt, Germany). Methylester of 5-doxyl-palmitic acid (MeFASL (10,3)) was synthesized by Professor S. Pečar (Faculty of Pharmacy, University of Ljubljana, Ljubljana, Slovenia).

### 4.2. Cell Culture

Cells were routinely maintained and passaged in Dulbecco’s modified Eagle’s Medium (DMEM) supplemented with 10% (*v*/*v*) heat inactivated foetal calf serum (HIFCS), 2 mM Glutamax^TM^-I, penicillin (100 U/mL) and streptomycin (100 μg/mL) at 37 °C in a humidified atmosphere of 5% (*v*/*v*) CO_2_. HEK-GnRH-R stable cell lines (HEK-GnRH-R and HEK-HAGnRH-R) were maintained in G418-containing medium (500 μg/mL). Previously published equilibrium dissociation constants (K_d_) and receptor number (B_max_) values for the HEK-GnRH-R stable cell line were 0.2 nM and 3.1 pmol/mg protein [26], K_d_ and B_max_ values for HEK-HAGnRH-R were 0.27 nM and 3.0 pmol/mg protein [33] and K_d_ and B_max_ values for the αT3-1 cells were 0.50 nM and 1.6 pmol/mg protein, respectively [25]. αT4 cells, a gonadotrope/thyrotrope progenitor derived cell line that do not express GnRH-R [54], and untransfected HEK-293 were used as corresponding controls for the αT3-1 and HEK-HAGnRH-R cells, respectively.

### 4.3. Confocal Microscopy

To assess the localization of GnRH-R within the PM lipid rafts of HEK-293 cells, we used the commercially available Vybrant^®^ Alexa Flour^®^ 488 Lipid Raft Labeling Kit. Fluorescent labeling of lipid rafts in live cells was performed at 4 °C according to the manufacturer’s instruction. Briefly, HEK-HAGnRH-R cells were plated on coverslides coated with 0.01% poly-D-lysine, which were individually placed into 60 mm Petri dishes containing 5 mL of complete DMEM. After 48 h, the cells were first serum-starved for 2h at 37 °C in HEPES-buffered DMEM and then treated with the GnRH-R agonist D-Trp^6^-GnRH (1 μM, 1 h, 37 °C) or methyl-β-CD (10 mM, 1 h, 37 °C), if required. The cells were subsequently washed once with chilled complete DMEM medium, and 250 μL of chilled Alexa Fluor 488 conjugate of cholera toxin subunit B (CT-B) (1 μg/mL) was added to each coverslide and the cells incubated for 10 min at 4 °C. CT-B conjugate binds to the pentasaccharide chain of PM ganglioside GM_1_, which selectively partitions into lipid rafts. After washing with chilled PBS, 250 μL of chilled anti-CT-B antibody was added to each coverslip to cross-link the CT-B lipid rafts, and the cells were then incubated for 15 min at 4 °C. After washing with chilled PBS, cells were fixed in 4% paraformaldehyde for 20 min at 4 °C. Fixed cells were then washed three times in PBS, PM permeabilized (PBS containing 0.01% Triton X-100), blocked (PBS containing 1% BSA) and incubated with a 1:50 dilution of primary rat anti-HA monoclonal antibody in blocking buffer overnight at 4 °C. The cells were then washed with PBS (three times) and incubated for 1 h at room temperature in a 1:50 dilution of TRITC-conjugated anti-rat IgG and then extensively washed. Cells were then mounted with an anti-fading ProLong^®^Gold reagent (Molecular Probes, Netherlands), sealed and examined under an oil immersion objective (Planapo 40×, N.A. = 1.25) using a Leica multispectral confocal laser microscope (Leica TCS NT, Heidelberg, Germany). Sequential detection of fluorescently labelled lipid rafts, HA-tagged GnRH-R and nuclei was achieved with the use of excitation laser lines at 488 nm (Argon), 543 nm and 633 nm (Helium-Neon), respectively. The fluorescence from the channels was collected sequentially and images produced with an 8-fold frame, averaging at a resolution of 1024 × 1024 pixels. Optical sections (1.0 µm) were taken and representative sections corresponding to the middle of the cells were presented using Adobe Creative Cloud (Adobe Inc., San Jose, CA, USA). The open-source image analysis software ImageJ (1.53c, National Institutes of Health, Bethesda, MD, USA) [55] was used to perform the colocalization analysis, and the Pearson’s correlation coefficient is reported to indicate the degree of colocalization. The values for Pearson’s correlation coefficient can range from +1 for perfect correlation, 0 for no correlation, and −1 for perfect anti-correlation.

### 4.4. Isolation of Detergent-Resistant Membranes (DRMs)

DRMs were isolated from HEK-293 cells labbeled with Vybrant^®^ Alexa Flour^®^ 488 Lipid Raft Labeling Kit as previously described [56]. Briefly, after being incubated with 1 mL of lysis buffer (0.5% Triton X-100/1 mM EDTA/1 mM Na_3_VO_4_/1 mM PMSF/10 μg/mL aprotinin/150 mM NaCl/25 mM Mes, pH 6.5) for 30 min at 4 °C, cells were homogenized, diluted with 1.0 mL of 85% sucrose, and loaded to the bottom of an ultracentrifugation tube. Samples were overlaid with 1.5 mL of 35% sucrose and 1.25 mL of 5% sucrose and spun 24 h at 200,000× *g* at 4 °C in a Beckman SW-41 rotor. DRMs were visible at the 35%/5% sucrose interface. Eight 400 μL fractions were collected from the top of the tube total fluorescence of each fraction was measured using a Mithras LB 940 multimode microplate reader (Berthold Technologies, Bad Wildbad, Germany).

### 4.5. Enzyme-Linked Immunosorbent Assay (ELISA)

An ELISA assay for the measurement of surface-expressed HA-tagged GnRH-R and quantification of receptor internalization was performed as described previously [33]. Briefly, cells were plated out at a density of 1.5 × 10^5^ cells per well in a 24-well plate. After 48 h, cells were treated as required in either HEPES-modified DMEM with 0.1% BSA, pH 7.4, or methyl-β-CD at 37 °C before fixing with 4% paraformaldehyde for 20 min at 4 °C. Cells were then washed three times in PBS, blocked (PBS containing 1% BSA) and incubated with a 1:600 dilution of primary rat anti-HA monoclonal antibody in blocking buffer overnight at 4 °C. Cells were subsequently washed with PBS (three times) and incubated for 1 h at room temperature in a 1:1000 dilution of HRP-conjugated anti-rat IgG and then extensively washed. The reaction was developed using the 3,3’,5,5’-tetramethylbenzidine (TMB) liquid substrate system, stopped after 10 min at 37 °C with 0.5 N H_2_SO_4_, and colorimetric measurement performed at 450 nm using a Rosys Anthos Reader 2010 (Anthos Labtec Instruments, Wals, Austria). Untransfected HEK-293 cells were assayed concurrently to determine the background. Determinations were made in triplicate. The obtained data were analyzed, and a graph was created using GraphPad Prism 8.0 computer software (La Jolla, CA, USA).

### 4.6. Intracellular Ca^2+^ Activity [Ca^2+^]_i_ Measurements

[Ca^2+^]_i_ measurements were performed as described previously [57,58] using a multimode microplate reader Mithras LB 940 (Berthold Technologies, Bad Wildbad, Germany). Briefly, trypsinized HEK-HAGnRH-R cells were resuspended in Dulbecco’s phosphate-buffered saline (DPBS) supplemented with CaCl_2_, MgCl_2_, glucose, and pyruvate to a density of 4 × 10^6^/mL and loaded with 2.5 μM fura-2/acetoxy-methyl ester (Fura-2-AM) dissolved in DMSO for 30 min at room temperature (22 ± 2 °C). After washing, the cells were resuspended in DPBS or DPBS containing 10 mM methyl-β-CD to a density of 2 × 10^6^/mL and cells were then seeded in black 96-well plates at a density of ~4 × 10^5^ cells/well. For an initial 40 s period (time resolution 0.5 s), the baseline fluorescence was determined by calculating the ratio of the fluorescence emission intensity obtained at 510 nm with excitation at 340 nm and at 380 nm (ratio F_340_/F_380_). Subsequently, D-Trp^6^-GnRH (final concentration 1 μM) was injected automatically, and again, the fluorescence intensity was measured for additional 180 s. All measurements were made at 30 °C and were performed within 2–3 h after Fura-2 AM loading. Control experiment was also performed with cells pretreated with 1 μM thapsigargin, a drug known to discharge the intracellular Ca^2+^ stores and changes in the ratio F_340_/F_380_ used as an index of variation in [Ca^2+^]_i_.

### 4.7. Electron Paramagnetic Resonance (EPR) Spectroscopy

EPR measurements were performed as described previously [51]. First, a thin film of the lipophilic spin probe MeFASL (10,3) (50 µL ethanol solution, C = 10^−4^ mol/L) was prepared on the wall of a glass tube by rotary evaporation of ethanol. αT4, αT3-1, HEK-293, HEK-HAGnRH-R or HEK-GnRH-R cells harvested under confluent conditions (~5 × 10^6^ cells) and suspended in 2 mL of HEPES-buffered DMEM, placed in a glass tube, and agitated manually for 10 min to allow the spin probe to penetrate the cell membranes. If required, cells were treated with either the GnRH-R agonist D-Trp^6^-GnRH (1 μM, 30 min, 37 °C) or methyl-β-CD (10 mM, 1 h, 37 °C) before labeling with the spin probe. The sample was then centrifuged (120× *g* for 3 min), and spin-labeled cells were transferred from the pellet into a 1-mm-diameter glass capillary tube for EPR measurement. Measurements were performed using a Bruker ESP 300 X-band EPR spectrometer (Bruker Analytische Messtechnik, Germany) at 20 °C (to prevent rapid reduction of spin probes to hydroxylamines), with a microwave frequency of 9.59 GHz, power set to 20 mW, modulation frequency of 100 kHz, and modulation amplitude of 0.2 mT. Each experiment was repeated at least four times.

### 4.8. Computer Simulation of EPR Spectra and GHOST Condensation Procedure

By computer simulation of the EPR spectra line shape of the spin probe, which is mainly distributed in the cell PM [31], in combination with the GHOST condensation procedure, information about PM domain structure and domain properties can be obtained [28]. The model takes into account that the local rotational motion is limited but fast with respect to the EPR time scale and that the EPR spectrum is a superposition of several spectral components reflecting different modes of rotational motion of spin probe molecules in different membrane regions. Each spectral component is described with different sets of spectral parameters, including the order parameter (S), the rotational correlation time (τc), the polarity correction factors of the hyperfine splitting tensor A and the g tensor (pA and pg), and a broadening constant (W) [28]. The order parameter (S) indicates the time-averaged deviation of the spin probe acyl chain from the perpendicular to the bilayer plane and is 1 for perfectly ordered structures and 0 for disordered isotropic motion of the molecules, the rotational correlation time (τc) describes the speed of motion of the spin probe in the membrane, and the polarity correction factor pA provides information about the polarity of the spin probe environment. In addition, the relative proportion of each spectral component (d) is determined, which describes the relative fraction of spin probes with a certain motion mode and depends on the distribution of the spin probe to different membrane regions. To obtain the best fit of the calculated EPR spectrum to the experimental one, a stochastic and population-based genetic algorithm was used in combination with Simplex Downhill (HEO) [36]. The computer simulation procedure is implemented in the software package EPRSIM, which includes multi-run HEO optimization in combination with the condensation procedure GHOST, which filters the solutions according to their goodness of fit and solution density in the parameter space and performs group detection by a slicing procedure [28]. It allows the determination of the number of distinct regions defined by S, τc, W, and pA in the membrane. Following this procedure, 200 independent HEO simulation runs were performed for each spectrum. All sets of parameters with the best fit obtained by 200 optimizations are summarized in two-dimensional cross-sectional plots: S-τc, S-W and S-pA diagrams; the other two parameters of each diagram are defined by the intensity of the colors: red, green, and blue for τc, W, and pA, respectively (GHOST diagrams) [28,59]. In addition, the corresponding errors are estimated by covariance matrix analysis, which determine deviations from the estimated mean [28].

### 4.9. Molecular Dynamics (MD) Simulations

The effect of cholesterol inclusion on properties of the membrane systems was also assessed by MD simulations. Ten membrane systems were used: SDPC (1-stearoyl-2-docosahexaenoyl-sn-glycero-3-phosphatidyl-choline), DOPC (1,2-dioleoyl-sn-glycero-3-phosphatidylcholine), DPPC (1,2-dipalmitoyl-sn-glycero-3-phosphatidylcholin), DSPC (1,2-distearoyl-sn-glycero-3-phosphatidylcholine) with (33%) and without cholesterol. Each system was built, solvated with explicit water molecules, neutralized, and its ionic strength was adjusted using the CHARMM-GUI builder [60]. Prior to production runs, the geometry of the system was optimized by energy minimization and further relaxed by a sequence of equilibration steps where harmonic positional restraints were applied to the lipid heads, and gradually released throughout the equilibration. Upon equilibration was completed, three independent trajectories of each system were spawned using a random seed. Production simulations for each replica were run in the NPT ensemble at 1013 bar and 310 K for 500 ns each. All simulations were run using ACEMD [61] and the CHARMM36 force field [62]. The first 50 ns of each trajectory were discarded in the analysis. Analysis of the membrane local properties was performed using the default parameters in MEMPLUGIN [41], to obtain the following biophysical properties in the simulated membranes: S_CD_ order parameter, membrane thickness, lipid interdigitation, and area per lipid.

### 4.10. Statistical Analysis

Statistical significance was determined using Student’s *t*-test when comparing the GnRH-R expressing cell line with the corresponding control or the one-way ANOVA followed by Bonferroni’s post hoc test when testing the effect of increasing concentrations of methyl-β-CD on GnRH-R surface expression and internalization. SigmaPlot 12.5 (Systat Software, Inc., Erkrath, Germany) was used for data analysis and GraphPad Prism 9.0 (GraphPad Software, San Diego, CA, USA) for plotting. Differences are considered significant at *p* < 0.05.

## Data Availability

Data is available from the authors.

## Supplementary Materials

The following are available online, Figure S1: Sucrose gradient centrifugation of HEK-293 cells labeled with Vybrant® Alexa Flour 488 Lipid Raft Labeling Kit, Figure S2: Comparison of PM properties of HEK-293 stable cell lines expressing HA-tagged and WT rat GnRH-R, Figure S3: Effect of agonist treatment on the PM properties of the HEK-HAGnRH-R cell line.
