# Supplementary material for: Electron Paramagnetic Resonance Gives Evidence for the Presence of Type 1 Gonadotropin-Releasing Hormone Receptor (GnRH-R) in Subdomains of Lipid Rafts
Source: Molecules. 2021 Feb 12;26(4):973. doi: 10.3390/molecules26040973 (PMC7918721; doi:10.3390/molecules26040973)
Supplement: Supplementary file 1 [file molecules-26-00973-s001.pdf]

Supplementary Materials

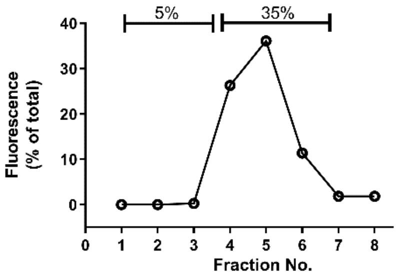

Figure 1. Sucrose gradient centrifugation of HEK-293 cells labeled with Vybrant® Alexa Flour 488 Lipid Raft Labeling Kit. Note the peak of the fluorescence at fractions 4–5 corresponding to the 5%/35% sucrose interface corresponding to raft-like PM.

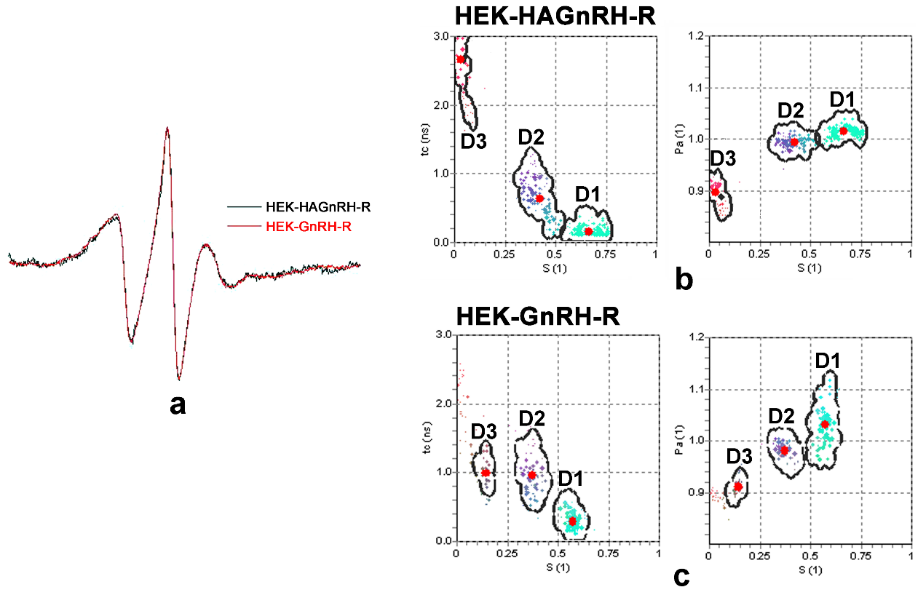

Figure 2. Comparison of PM properties of HEK-293 stable cell lines expressing HA-tagged and WT rat GnRH-R. (**a**) The EPR spectra of lipophilic spin probe MeFASL (10,3) in the PM of the HEK-HAGnRH-R stable line expressing HA-tagged GnRH-R (black line) and HEK-GnRH-R stable cell line expressing WT GnRH-R (red line). The spectra are the sum of the spectra of at least four independent experiments. (**b**,**c**) S-τc (order parameter vs. rotational correlation time) and S-p_A (order parameter vs. polarity correction factor) GHOST diagrams of EPR spectral parameters of motional modes of spin probe in the PM of HEK-HAGnRH-R (b) and HEK-GnRH-R cells (c). Red dots are the average values of spectral parameters obtained from multiple runs of the HEO optimization procedure, which give the best fits to the experimental spectra. Borders of each group determine the interval in which the solutions can be obtained. GHOST diagrams are representative of at least four independent experiments. Domain 1 (D1), domain 2 (D2), domain 3 (D3).

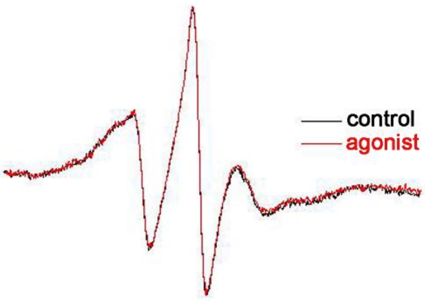

Figure 3. Effect of agonist treatment on the PM properties of the HEK-HAGnRH-R cell line. Comparison of EPR spectra of lipophilic spin probe MeFASL(10,3) in the PM of untreated (control; black line) and agonist treated (1 μM D-Trp^6-GnRH; 30 min at 37 °C; red line) HEK-HAGnRH-R cells. The spectra are the sum of the spectra of at least four independent experiments.
